# Better Rooting Procedure to Enhance Survival Rate of Field Grown Malaysian Eksotika Papaya Transformed with 1-Aminocyclopropane-1-Carboxylic Acid Oxidase Gene

**DOI:** 10.5402/2013/958945

**Published:** 2012-10-21

**Authors:** Rogayah Sekeli, Janna Ong Abdullah, Parameswari Namasivayam, Pauziah Muda, Umi Kalsom Abu Bakar

**Affiliations:** ^1^Department of Microbiology, Faculty of Biotechnology & Biomolecular Sciences, Universiti Putra Malaysia, Selangor Darul Ehsan, 43400 UPM Serdang, Malaysia; ^2^Department of Cell & Molecular Biology, Faculty of Biotechnology & Biomolecular Sciences, Universiti Putra Malaysia, Selangor Darul Ehsan, 43400 UPM Serdang, Malaysia; ^3^Malaysian Agricultural Research and Development Institute (MARDI), P.O. Box 12301, 50774 Kuala Lumpur, Malaysia

## Abstract

A high survival rate for transformed papaya plants when transferred to the field is useful in the quest for improving the commercial quality traits. We report in this paper an improved rooting method for the production of transformed Malaysian Eksotika papaya with high survival rate when transferred to the field. Shoots were regenerated from embryogenic calli transformed with antisense and RNAi constructs of 1-aminocyclopropane-1-carboxylic acid oxidase (ACO) genes using the *Agrobacterium tumefaciens*-mediated transformation method. Regenerated transformed shoots, each measuring approximately 3-4 cm in height, were cultured in liquid half-strength Murashige and Skoog (MS) medium or sterile distilled water, and with either perlite or vermiculite supplementation. All the culturing processes were conducted either under sterile or nonsterile condition. The results showed that rooting under sterile condition was better. Shoots cultured in half-strength MS medium supplemented with vermiculite exhibited a 92.5% rooting efficiency while perlite showed 77.5%. The survival rate of the vermiculite-grown transformed papaya plantlets after transfer into soil, contained in polybags, was 94%, and the rate after transfer into the ground was 92%. Morpho-histological analyses revealed that the tap roots were more compact, which might have contributed to the high survival rates of the plantlets.

## 1. Introduction 

The world production of papaya in 2008 was valued at 9.1 million tonnes [1], and Malaysia is listed as one of the top six major exporters. The most popularly grown cultivar and the main variety grown for export is Eksotika, which is a high yielding variety producing 50–70 tonnes per hectare over a two-year crop cycle [2]. The release of Eksotika papaya variety in 1987 had since resulted in an increase in its production areas in Malaysia. Due to its importance to Malaysian economy, some important traits in Eksotika papaya need to be further improved to increase the papaya production so as to meet and widen the market demand. Similar to other tropical fruits, Eksotika fruit has a very short shelf life. There is a need to extend its shelf life in order to reduce post-harvest losses and to increase its export potential to distant destinations. Throughout the years, attempts had been made through conventional breeding and manipulation of post-harvest approaches but all were unsuccessful in improving this trait. Increasing the shelf life of papaya fruit through genetic engineering is therefore a more promising option. The ability to delay the ripening process in the fruits will result in fresher and more nutritious papayas, which are also less prone to damages during handling and transportation. 

Critical prerequisites in the development of transgenic papaya include efficient and reproducible plant transformation and regeneration systems, and a successful acclimatization process of the transgenic plants for field transfer [3]. The two major problems faced in developing transgenic Malaysian Eksotika papaya plants are low rooting efficiency of regenerated shoots and low acclimatization rate of rooted transgenic papaya plants in the field. These problems could probably be due to poor quality roots such as thickened, callused, and absent lateral roots and root hairs formation, which all affect the uptake of nutrients by the plants after transfer into soil. Therefore, rooting efficiency and quality roots formation are critical in ensuring successful and continuous production of transgenic Eksotika papaya. Indole-3-butyric acid (IBA) had been reported by many researchers as the best auxin for root induction in papaya [4] at an optimum concentration of 10 *μ*M [5]. IBA was known to have greater ability to promote rooting with less callus formation. Numerous authors reported that rooting in agar medium produced thick, short, and stumpy roots [6–10]. Many authors reported that vermiculite was able to improve rooting efficiency and produce good quality roots which were finer with abundant lateral branches and root hairs [11, 12]. 

The aim of the study reported here was to improve the rooting efficiency and roots quality of tissue-cultured Malaysian Eksotika papaya shoots regenerated from embryogenic calli transformed separately with RNAi and antisense constructs of the ACO genes (*ACO*1 and *ACO*2). Vermiculite was used as a substrate for rooting the *in vitro* papaya shoots under two different culture conditions (sterile versus non-sterile). Perlite was also used as an alternative substrate for comparison. The number of roots produced, average root length obtained, and the survival rate of the rooted shoots during acclimatization process were determined. PCR positive plantlets produced from transformation events using the respective gene construct were used for the rooting studies. Morphological and histological analyses were carried out to assess the quality of the roots produced. The improved rooting method presented here will be useful for the development of transgenic papaya plants facing rooting problem (poor root quality) after a genetic manipulation event.

## 2. Materials and Methods

### 2.1. Antisense and RNAi Constructs Development

An antisense gene cassette, designated as pASACO2E1, was constructed by inserting the PCR-amplified 1441 bp full-length *ACO*2 in an antisense orientation into the t-DNA region of the binary vector pGA643, which harbours an *npt*II selectable marker gene, a *CaMV*35*S* promoter, and a *nos* terminator. PCR amplification of the *ACO*2 gene was done using specific primers as shown in Table 1. The PCR reaction mixture consisted of 1X PCR buffer, 25 mM MgCl_2_, 2 mM dNTPs, 10 *μ*M each of the sense and antisense primers, 3 U Taq polymerase, and 300 ng of plasmid DNA, and the final volume was adjusted to 20 *μ*L with sterile distilled water. The PCR amplification condition was as shown in Table 2.

The pOpOff 2 vector [13] was used for all the three RNAi constructs, designated as pRNAiCACO, pRNAiACO1, and pRNAiACO2. This pOpOff 2 vector contains an *npt*II selectable marker gene, a *gus*A reporter gene, an inducible *pOp*6 promoter, a *CaMV*35*S* promoter, and a *nos* terminator. This inducible RNAi vector was obtained through an MTA agreement between the Malaysian Agricultural Research and Development Institute (MARDI) and the Commonwealth Scientific and Industrial Research Organization (CSIRO) for use in this research. The pRNAiACO1 and pRNAiACO2 constructs contain the 3′UTR region of the respective *ACO*, and the pRNAiCACO contains the conserved regions of both genes. The primers used for each gene constructs were designed using the Pearl Primer software (Sourceforge.net). List of the primers, their sequences, and the expected amplicons size are as shown in Table 1. The same PCR reaction mixtures as described earlier were used for the PCR amplification. The PCR products obtained from the target regions were individually cloned into an intermediate vector, PCR8/GW/TOPO, prior to subcloning into the pOpOff 2 vector using the Gateway directed recombination system (Invitrogen, California, USA). 

### 2.2. Induction of Embryogenic Callus

Embryogenic calli of papaya (*Carica papaya *var. Eksotika) were initiated from immature zygotic embryos obtained from Malaysian Eksotika papaya fruit of 90 days after pollination. The immature embryos were cultured on an induction medium consisting of half-strength MS [14] basal salts supplemented with 50 mg/L myo-inositol, full-strength MS vitamins, 6% (w/v) sucrose, 45.2 *μ*M 2,4-dichlorophenoxyacetic acid (2,4-D), and 0.35% (w/v) phytagel. The pH of the medium was adjusted to 5.8 prior autoclaving. The callus cultures were grown at 25 ± 2°C in the dark for one month.

### 2.3. Papaya Transformation and Shoot Regeneration

One-month-old Eksotika embryogenic calli were transformed separately with the antisense construct (pASACO2E1) and the RNAi constructs (pRNAiACO1, pRNAiACO2, and pRNAiCACO) using a previously established *Agrobacterium-*mediated transformation method for Eksotika [15]. The *Agrobacterium* strain LBA 4404 was used in all the transformation events. To select for putative transformed tissues, the transformed calli were transferred onto half-strength MS medium supplemented with kanamycin. The selection process was carried out for a total of four months. The first selection was carried out with 75 mg/L kanamycin for one month, followed by 150 mg/L kanamycin for the remaining 3 months. Surviving calli on the selection media were transferred onto the De Fossard (DF) [16] regeneration medium supplemented with 0.89 *μ*M 6-benzyladenine (BA), 1.1 *μ*M 1-napthaleneacetic acids (NAA), and 150 mL young coconut water for shoot regeneration. In order to minimize potential accumulation of ethylene inside the culture flask, the mouth of each culture flask was covered with 3 pieces of tissue paper. Following which a piece of aluminium foil, punched with holes measuring 5 mm each, was placed over the tissue papers. This setup was maintained in all subculturing processes and was removed during the initial acclimatization step. In this study, vancomycin and cefotaxime at 100 and 150 mg/L, respectively, were used during the selection and regeneration processes and were subsequently removed during the rooting process. Individual shoots from a shoots cluster were excised and subcultured on the same but fresh DF medium prior to transferring onto the rooting medium.

### 2.4. PCR Analysis of Putative Transformants

The presence of the transgene(s) in transformed regenerated papaya shoots was verified by PCR using the TPersonal Thermocycler (Biometra GmbH, Goettingen, Germany). Genomic DNA of the papaya leaves were extracted using the Qiagen kit (Qiagen, Hilden, Germany) with a starting material of 100 mg for each sample. A total of 50 ng of each extracted genomic DNA samples was used for the PCR analyses. Putative transgenic plants harbouring the antisense transgenes were verified by amplifying the antisense *ACO*2 and *npt*II genes. For putative transgenic shoots harbouring the RNAi transgenes were likewise verified for the presence of the *npt*II, *gus*A, PDK intron, *ACO*1, and *ACO*2 genes. List of primers used are as shown in Table 3. 

The same PCR reaction mixture and PCR cycling conditions were used as described earlier in the constructs development section. Each PCR amplified products were subjected to 1% (w/v) agarose gel electrophoresis, and bands were visualized under UV light and photographed using the Gel Doc XR System equipment (Bio Rad, Corston, United Kingdom). All PCR-positive candidates were selected for the rooting experiments. The 4-cm (height) PCR-positive regenerated shoots were transferred and cultured on half-strength MS solid medium for 4 weeks at 25 ± 2°C before they were used in the rooting experiments.

### 2.5. Roots Induction Using DF Medium

A previously established rooting method [15] was also conducted in this study for comparison. Individual PCR-positive shoots, about 4-cm in height, were excised and sub-cultured on half-strength DF medium for 4 weeks before they were used for the rooting experiment. After 4 weeks, the individual shoots were transferred to a root initiation medium consisted of full-strength DF medium supplemented with 4.9 *μ*M indole-3-butyric acid (IBA). After 3 days on this root initiation medium, the shoots were transferred to full-strength basal DF medium supplemented with 10 *μ*M riboflavin. A total of 180 positive plantlets were transferred to this rooting medium which comprised of 40 plantlets for each RNAi constructs and 60 plantlets for the antisense construct. The cultures were maintained at 25 ± 2°C for 8 weeks under a 16-hour photoperiod supplied by white fluorescence light at 25 mMol photon/m^2^/s and observed for roots development. Sub-culturing to fresh media was done at 4-week intervals until roots were observed.

### 2.6. Effects of Different IBA Concentrations on Roots Development of Putative Transgenic Papaya Shoots Using MS Medium

Individual PCR-positive shoots of approximately 4-cm in height were excised from shoot clusters and sub-cultured on half-strength MS medium for 4 weeks before they were transferred onto rooting medium consisted of full-strength MS medium supplemented with IBA at 4.9, 9.8, and 14.8 *μ*M, respectively. The cultures were kept for 4 days in the dark at 25 ± 2°C. After 4 days on the MS medium containing different concentrations of IBA, the shoots were transferred to full-strength MS medium supplemented with 10 *μ*M riboflavin, and observed for roots development. The cultures were maintained at 25 ± 2°C for 8 weeks under a 16-hour photoperiod of white fluorescence light supplied at 25 mMol photon/m^2^/s. The number and length of roots formed, and the quality of the roots were recorded after 8 weeks of culture. The experiment was replicated thrice with 20 explants per replicate. 

### 2.7. Effects of Different Rooting Substrates on Roots Development of Putative Transgenic Papaya Shoots Using MS Medium

Positive transgenic shoots that reached 4 cm in height were first cultured on full-strength MS solid medium supplemented with 9.8 *μ*M IBA for 4 days before they were transferred to the respective sterile rooting medium consisting of either half-strength liquid MS medium or sterile distilled water, and each medium was supplemented with either vermiculite or perlite (to replace the gelrite in the MS medium). All the shoots were then allowed to grow for 4 weeks. For half-strength liquid MS medium, only sterile condition was applied to avoid contaminating the cultured shoots. For treatment in distilled water medium, two different culturing conditions were applied, sterile and non-sterile conditions. A sterile condition implied that all the cultures were contained in sealed jam jars. While in non-sterile condition, the mouth of the jam jar was covered with a clear plastic sheet punched with 22 holes, each hole measuring 5 mm diameter. All the cultures were likewise kept at 25 ± 2°C under a 16-hour photoperiod of white fluorescence light supplied at 25 mMol photon/m^2^/s. The number and length of roots formed, and the quality of the roots were recorded after 4 weeks of culturing. The experiments were repeated thrice with 20 explants used per treatment. 

### 2.8. Acclimatization and Field Planting of Rooted Putative Transgenic Papaya Plantlets

The best rooting method, which is half-strength liquid MS medium supplemented with vermiculite, was used for subsequent rooting of the putative transgenic papaya plants. Rooted plantlets with finer roots system, consisting of at least 4 lateral branches and abundant root hairs, were individually transferred to 9 cm × 15 cm size polybags containing vermiculite, sand, and mixed soil (soil and coconut husk) at a ratio of 1 : 1 : 1. Each polybag plantlet was then covered with a clear plastic sheet punched with 22 holes, and left in a temperature controlled growth chamber at 25 ± 2°C for 2 weeks. A total of 200 plantlets were transferred to the polybags. After 2 weeks, the plantlets were repotted to bigger polybags measuring 14 cm × 26 cm, and grown in a temperature controlled chamber at 28 ± 2°C for 3 weeks. Following which the acclimatized plantlets (in the polybags) were moved to the transgenic glasshouse without temperature control for further hardening. Throughout the study, the temperature inside the transgenic glasshouse was about 32 ± 2°C with approximately 90% light transmission and a relative humidity between 70–90%. The survival rates of the plantlets were recorded after 4 weeks of transfer to this transgenic glasshouse. Subsequently, sixty of the plants were transferred into soil, with a planting distance of 2.2 m between rows and 2 m within each row, in a netted field measuring approximately 24 m × 18 m. The survival rates of the plants were recorded after 8 weeks. Each plant received 8 L of water per day or whenever required. The plants were fertilized monthly with NPK green fertilizer (NPK fertilizer with 1 : 1 : 1 ratio of nitrogen : phosphorus : potassium). Pest and disease controls were applied whenever necessary. 

### 2.9. Histological Studies on Roots Produced from Agar-, Vermiculite-, and Perlite-Grown Plantlets

Roots produced from gelrite agar-, vermiculite-, and perlite-grown plantlets were subjected to histological analysis to determine the morphological differences between the types of root formed. For the histological analysis, sample fixation, processing, and staining were performed at the Microscopy Laboratory, Advanced Biotechnology and Breeding Centre, Malaysian Palm Oil Board (MPOB), Selangor Darul Ehsan, Malaysia. The root samples were fixed in FAA solution (0.2 *μ*M phosphate buffer, 10% (w/v) paraformaldehyde, 25% (v/v) glutaraldehyde) and 1% (w/v) caffeine at room temperature for 48 hours. After fixation, the tissues samples were dehydrated through a series of ethanol: 30% (v/v) for 30 min, 50% (v/v) for 45 min, 70% (v/v) for 45 min, 89% (v/v) for 60 min, 90% (v/v) for 60 min, 95% (v/v) for 60 min, and finally twice in absolute ethanol for 60 min each. Subsequently, the tissues were embedded in Technovit 7100 resin (Heraeus, Hanau, Germany) and sectioned at 3.5 *μ*m thicknesses. The sections were stained with 0.05% (w/v) toluidine blue and viewed under a Nikon microscope multizoom AZ100 (Nikon, Melville, New York, USA). Six slides of each sample for every section were observed. 

### 2.10. Statistical Analysis

The data expressed were analyzed using the one-way ANOVA using the statistical analysis software (SAS) (version 8.0, SAS Institute Inc., Cary, North Carolina, USA). 

## 3. Results and Discussion

### 3.1. *Agrobacterium*-Mediated Transformation of Antisense and RNAi Constructs of 1-Aminocyclopropane-1-Carboxylic Acid Oxidase (ACO) Genes

In papaya transformation, immature embryos are commonly used for callus induction where a high percentage of zygotic embryos produced embryogenic callus or sometimes direct somatic embryos were formed on the apical domes of the immature embryos [15]. The production of embryogenic callus is faster compared to the mature seed-derived callus. However, the use of immature embryo was very much dependent on seasonal factor. In this study, embryogenic callus was induced by culturing immature zygotic embryos of Eksotika papaya, obtained at 90 days after pollination, on the induction medium supplemented with 45.2 *μ*M 2,4-D. 2,4-D was used only at the early stages of callus induction and selection of the transformants and was removed during the regeneration process. The zygotic embryos started to dedifferentiate after 7 days of culture, and after 4 weeks, embryogenic calli were observed. The *Agrobacterium *that harboured the respective antisense and RNAi constructs was used to infect calli induced from the immature Eksotika papaya embryos. A total of 15,000 calli and 6,000 calli were transformed with the RNAi and antisense constructs, respectively. Selection of the putative transformed tissues was carried out for 4 months on MS medium containing 75 mg/L kanamycin for 1 month, followed by 3 months with 150 mg/L kanamycin. 

**Figure 1:**
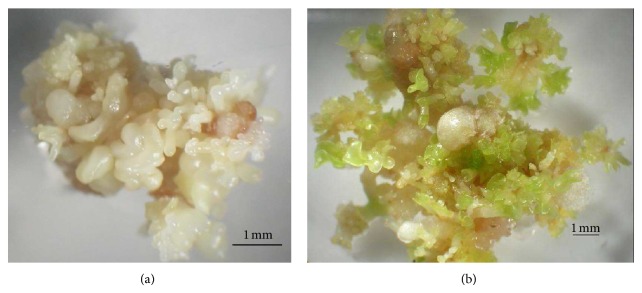
Embryogenic calli of Eksotika papaya. (a) Mature somatic embryos developed on De Fossard regeneration medium and (b) Regeneration of putative transformed calli.

The results showed that at 75 mg/L kanamycin, 90% of the calli survived and remained yellowish. For the first 2 weeks, calli subjected to 150 mg/L kanamycin were yellowish, and untransformed calli started to brown after 4 weeks. The surviving calli on the selection medium produced somatic embryos after culturing on the DF [16] regeneration medium supplemented with 0.89 *μ*M BA and 1.1 *μ*M NAA, as shown in Figure 1. On this medium, more than 98% of the putative transformed calli shooted. Approximately 10–15 shoots were obtained from each transformed callus. This showed that BA and NAA were suitable for the papaya cultures. BA was known to play an important role in breaking apical dominance and initiating axial shoots while NAA enhanced meristem cells elongation [17]. Coconut water added to the regeneration culture medium maintained healthier and greener shoots during the regeneration process of the transformed tissues. The beneficial effects of using coconut water had been reported in many different plants species [18].

### 3.2. PCR Analysis of Putative Transgenic Shoots

**Figure 2:**
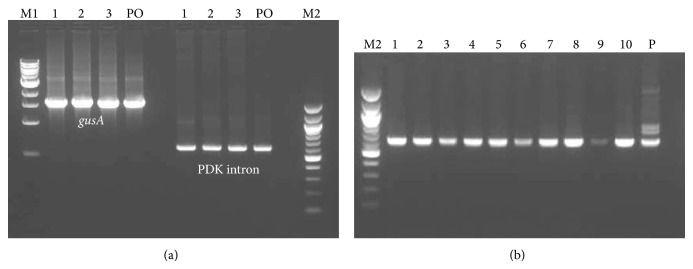
PCR analysis of transformed, regenerated shoots. (a) PCR analysis of genomic DNA obtained from putative transformants, harbouring the RNAi constructs, using *gus*A and PDK intron primers amplifying 1711 and 622 bp products, respectively. L1–L3 are putativetransgenic shoots, and (b) PCR analysis of antisense putative transgenic papaya plants using *npt*II primers producing a 610 bp product. L1–L10 are putative transgenic shoots. M1 and M2 represent 1 kb and 100 bp DNA ladders, respectively (Biolabs, California, USA), PO is the pOpOff 2 vector, and P is the pASACO2E1 plasmid as a positive control.

PCR analysis was carried out on single shoots of the transformed papaya lines (Figure 2). The results showed that out of 60 putative calli transformed with pASACO2E1, 46 were positives for *npt*II and *ACO*2. For RNAi constructs (pRNAiACO1, pRNAiACO2, and pRNAiCACO), 160 out of 176 lines tested positive for *npt*II, *gus*A, PDK intron, *ACO*1, and *ACO*2. All these PCR-positive shoots were used for the rooting experiment.

### 3.3. Rooting of Putative Transgenic Shoots Using DF Medium

*In vitro* rooting and survival of transformed plantlets after transfer into soil is a major problem in developing transgenic Malaysian Eksotika papaya variety. More than two decades ago, Winnaar [19] reported that rooting was crucial and posed to be a difficult stage in the micropropagation of papaya. Using a previously developed method [15], the PCR-positive regenerated shoots were transferred and cultured on half-strength basal salts DF solid medium for 4 weeks before they were used for the rooting experiments. Roots initiation was carried out on the DF medium supplemented with 4.9 *μ*M IBA, and the cultures were kept in darkness for 3 days before transferring to hormone free DF medium.

Roots started to form after 4 weeks on the DF basal medium. Out of 180 putative transgenic lines (40 plants for each RNAi constructs and 60 for pASACO2E1 antisense construct) transferred to the rooting medium, only 40 (22%) rooted after 8 weeks, as summarized in Table 4. The overall results showed that the DF medium was not suitable for the putative transgenic papaya plantlets. Quality of the roots produced was not consistent; some shoots produced good quality roots with abundant primary and lateral roots while some produced short stumpy and vitrified primary roots with sparsely distributed lateral roots. Problems of *in vitro* plants exhibiting low root quality, which were thick, callused, and lacking lateral roots and root hairs, when rooted on agar medium, had been previously reported [4, 6, 7]. Teo and Chan [9] revealed that roots growing on agar surface had normal morphology but roots that grew into the agar medium were thick and stumpy. In the current study, IBA at 4.9 *μ*M was applied to induce roots formation as it is widely used for papaya root induction worldwide due to less toxicity and more stability in comparison to naphthalene acetic acid (NAA). It was previously reported that shoots maintained too long in a culture medium containing IBA were stunted and exhibited thickened roots, and the shoots had more tendencies to produce calli rather than roots [4, 9]. In the current study, riboflavin was added to the rooting medium after the initiation phase to reduce the potential toxic effects of IBA. Drew et al. [5] reported that the effects of excess IBA was reduced when riboflavin was added as riboflavin rapidly photooxidised any IBA carried over to the hormone-free medium under light incubation. Based on root development, it showed that IBA was able to nullify the effect of IBA.

### 3.4. Effects of Different Concentrations of IBA on Roots Development in Putative Transgenic Eksotika Papaya Shoots Cultured on MS Medium

Improvement of roots quality in putative transgenic papaya shoots as a way to overcome the low survival rate of rooted shoots during acclimatization was done using MS medium to replace the DF medium. Individual shoots of approximately 3-4 cm in height were excised from shoot clusters and transferred to half-strength MS basal medium for 4 weeks prior to use in the rooting experiment. In the rooting experiment, the shoots were first pretreated in MS medium containing different concentrations of IBA (4.9, 9.8, and 14.8 *μ*M) for 4 days. 

**Figure 3:**
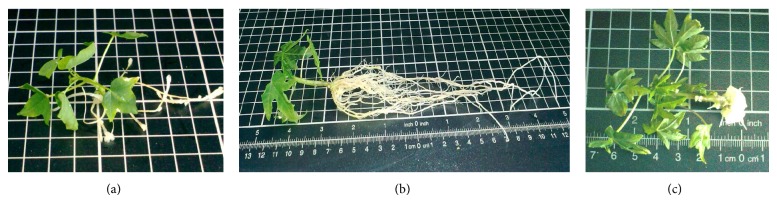
Effects of different IBA concentrations on rooting. (a) 4.9 *μ*MIBA, (b) 9.8 *μ*M IBA, and (c) 14.8 *μ*M IBA.

In this MS medium, it was observed that the roots were produced in a shorter time span compared to the original protocol using the DF medium (summarized in Table 4 and shown in Figure 3). Numerous researchers also favoured using MS as a rooting medium for papaya [11, 12, 20, 21]. In this study, some of the shoots were able to root after three weeks of culture, and the roots were initiated from the base of the shoots. Treatment with 4.9 *μ*M and 9.8 *μ*M IBA showed no significant differences (*P* < .05) in the rooting percentages. However, treatment with 4.9 *μ*M IBA produced fewer primary roots compared to 9.8 *μ*M IBA. It was also observed that more than 90% of the shoots cultured on 14.8 *μ*M IBA did not root even after 4 weeks, instead callusing was favoured. Treatment with 4.9 *μ*M and 9.8 *μ*M IBA produced good quality roots whereas treatment with 14.8 *μ*M IBA produced thick, stumpy, and vitrified roots. MS medium supplemented with 9.8 *μ*M IBA seemed to be the optimal concentration for stimulating roots formation in Malaysian Eksotika papaya, showing an increment of 50% rooting efficiency with many branching lateral roots. Therefore, 9.8 *μ*M IBA was used for all the subsequent experiments in this study.

### 3.5. Effects of Different Rooting Substrates on Root Development of Putative Transgenic Papaya Shoots Cultured on MS Medium and Distilled Water

Further rooting improvement involved using two different substrates (vermiculite and perlite) supplemented in half-strength liquid MS or in distilled water. The results were as summarized in Table 5. Plantlets that were grown in rooting medium consisting of sterile half-strength MS were healthier and grew more vigorously with green leaves compared to those grown in distilled water. Half-strength MS supplemented with vermiculite was shown to be a more suitable medium for rooting the putative transgenic Eksotika papaya shoots. Under this treatment, the roots started to form only after 10 days of transfer. Approximately 92.5% of the shoots rooted and exhibited better roots quality comprising of many lateral roots and root hairs with the mean root number per shoot and average root length of 4.8 and 4.2 cm, respectively. The plantlets also exhibited less percentage of leaves abscission. With perlite, rooting was delayed to after 15 days of transfer. A lower percentage of rooting (77.5%) was observed, and the roots produced were hard and compact with fewer numbers of lateral roots and root hairs. 

Different results were observed when the putative transgenic shoots were transferred under sterile versus non-sterile conditions to the rooting medium supplemented with distilled water. The shoots transferred under sterile condition grew healthier and greener, and leaf abscission was reduced. Under non-sterile condition, most of the leaves abscised after 5 days of transfer particularly those shoots grown in perlite supplemented rooting medium. Under this condition too, more than 75% of the leaves turned yellowish after 10 days of transfer and leaves abscission followed subsequently. Rooting was delayed to after 26 days of transfer. We attributed the poor shoots growth to insufficient nutrients supply from the water medium. 

### 3.6. Acclimatization of Putative Transgenic Plantlets Using DF Rooting Method

Papaya plants are susceptible to root rot and therefore acclimatization of papaya plants required potting mixtures that allow plenty of aeration [22]. It was reported previously [10, 20] that vermiculite was a good substrate for acclimatization of papaya plants. However, in this study, when the rooted papaya plantlets obtained using DF rooting method were transferred to the soil medium consisting of vermiculite, sand, and mix soil at a 1 : 1 : 1 ratio, they exhibited low survival rates (10 out of 40 rooted plantlets transferred survived). This may be due to poor roots quality that resulted in poor adaptation during acclimatization. We also observed that plantlets with short, stumpy roots did not survive when transferred into the soil medium. Such observations were consistent with [9, 23] who reported low survival rates for their plantlets bearing similar root morphologies. In this study, only 9 putative transgenic lines survived after transferred into soil in the field. Due to this low rooting efficiency and survival rate, it was therefore critical to improve both aspects in order to ensure successful transfer of larger number of transgenic plants to the field to allow field evaluations.

### 3.7. Acclimatization of Rooted Transgenic Plantlets in Half-Strength MS with Vermiculite

**Figure 4:**
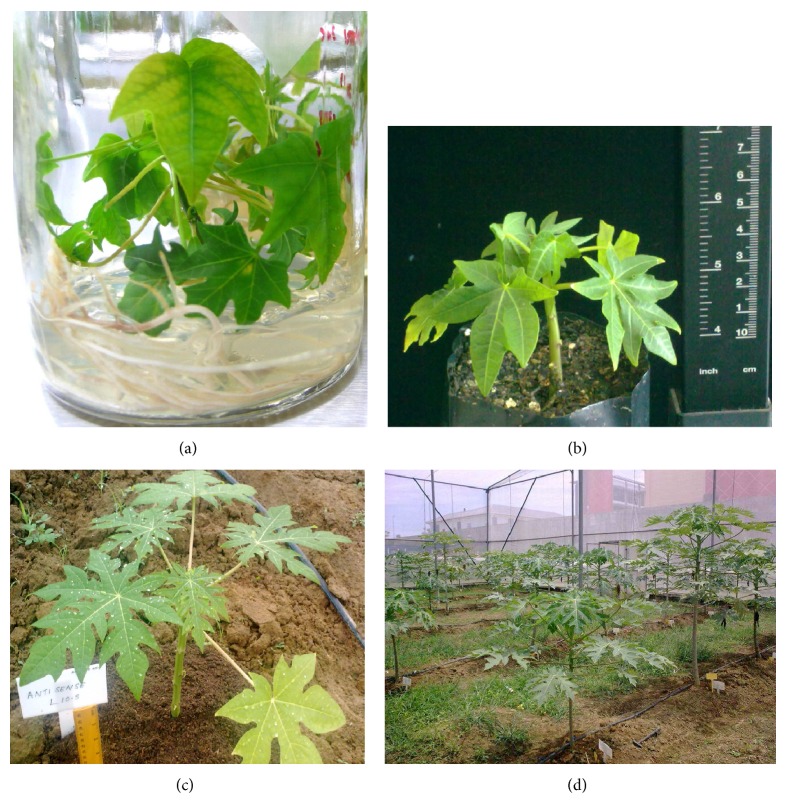
Acclimatization of putative transgenic plants using improved rooting method. (a) Rooted plantlet in an agar medium, (b) plantlet in soil mixture after 4 weeks, (c) putative transgenic plant after 3 weeks in soil in a net house, and (d) putative transgenic plants after 3 months in soil in a net house.

During the hardening process in the transgenic glasshouse, from a total of 200 rooted putative transgenic plantlets obtained using half-strength MS supplemented with vermiculite, more than 94% (188 plantlets) survived after 4 weeks. Out of sixty transgenic plantlets transferred into soil in the net house, more than 92% (55 plantlets) survived with healthy green leaves. We attributed such encouraging increase in survival rates to the better root quality produced. The roots produced in vermiculite medium were more compact compared to the roots produced in agar medium. Morphological analyses were also carried out on the T_0_ transgenic plants and wild-type plants using quantitative characters such as plant height, stem girth, height of first flower, height of first fruit, and mean internodes length of each surviving plantlet (results are summarized in Table 6 and Figure 4). In terms of growth performance, the non-transformed plantlet (control) grew more vigorously than the putative transgenics. 

### 3.8. Histological Analysis of Roots Produced

**Figure 5:**
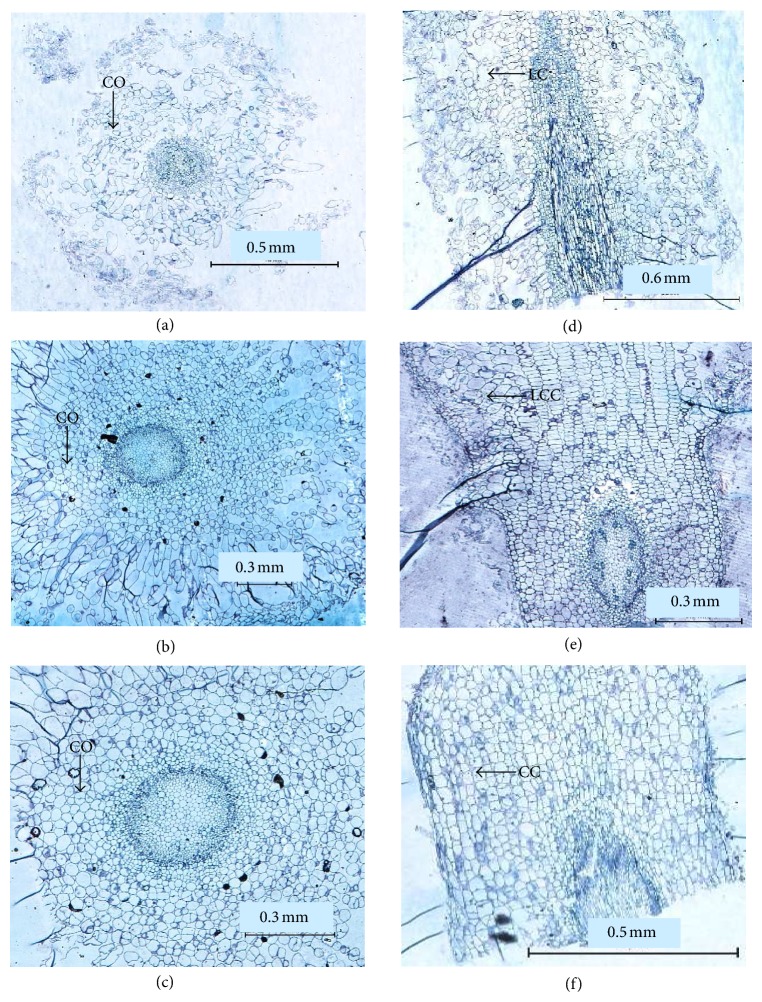
Histological analysis of roots of PCR-verified, transformed papaya plants grown in different rooting media. (a) to (c) are cross-sections of the roots, (d) to (f) are longitudinal-sections. (a) Agar, (b) vermiculite, (c) perlite, (d) agar, (e) vermiculite, and (f) perlite. CO signifies the cortex region, LC for loose cells, LCC for less compact cell, and CC for compact cells.

Histological analysis was carried out on roots produced from different rooting media: half-strength MS agar medium, and half-strength liquid MS supplemented with either vermiculite or perlite. Although both vermiculite and perlite were able to increase the rooting efficiency of transformed papaya, different root morphologies were observed (Figure 5). The roots produced in agar were thicker and vitrified (Figures 5(a) and 5(d)) compared to root produced in perlite and vermiculite. The cortex cells in perlite and vermiculite grown plants had distinct shapes and were regularly arranged. However, the roots produced in perlite medium were more compact with fewer lateral roots. We attributed such compactness to the closely arranged cortex cells (Figures 5(c) and 5(f)). The opposites were observed with roots grown in vermiculite medium (Figures 5(b) and 5(e)) where the cortex cells are less compact.

## 4. Conclusions 

In this study, an improved and efficient rooting method was established for the regenerated putative transgenic Malaysian Eksotika papaya shoots. The rooting percentage achieved was 92.5% using half-strength liquid MS mixed with vermiculite, with a survival rate of 92% after transfer into soil. This improved rooting system will be useful for the development of transgenic Malaysian Eksotika papaya with improved commercial traits.

## Figures and Tables

**Table 1:** Primers used for developing antisense and RNAi constructs.

Construct	Primer Code	Primer Sequence (5′-3′)	Length (bp)	Amplicon size (bp)
pASACO2E1	AS-ACO2F	gctctagacaaacaccaccaccagg	25	1441
	AS-ACO2R	cccaagcttacagaaacccaccaaag	26	

pRNAiACO1	pRNAiACO1F	cttctttctacaaccccagc	20	350
	pRNAiACO1R	gacaccgttttcccacact	19	

pRNAiACO2	pRNAiACO2F	tctactgtaactcctggtgc	20	317
	pRNAiACO2R	caccaaaggccactagacag	20	

pRNAiCACO	pRNAiCACOF	atgaaggagtttgcagtggg	20	340
	pRNAiCACOR	ccgttagtaatcagctcaag	20	

**Table 2:** PCR amplification conditions for antisense and RNAi constructs.

Construct	Predenaturation	Denaturation	Annealing	Primer extension	Final extension	PCR cycle
pASACO2E1	2 min, 94°C	45 sec, 94°C	1 min, 62°C	30 sec, 72°C	10 min, 72°C	35

pRNAiACO1	3 min, 94°C	1 min, 94°C	45 sec, 60°C	1 min, 72°C	10 min, 72°C	35
pRNAiACO2						
pRNAiCACO						

**Table 3:** Primers used for analysis of antisense and RNAi constructs in transformed papaya shoots.

Name of construct	Primer code	Primer sequence (5′-3′)	Length (bp)	Amplicon size (bp)
	*npt*IIF	ccttatccgcaacttctttacc	22	610
Antisense	*npt*IIR	caccatgatattcggcaagcag	22	
(pASACO2E1)	643F	actgacgtaagggatga	17	1700
	643R	tacattgccgtagatga	17	

	*npt*IIF	ccttatccgcaacttctttacc	22	610
	*npt*IIR	caccatgatattcggcaagcag	22	
	*gus*AF	catggtacgtcctgtagaaacc	22	1711
	*gus*AR	gaagatccctttcttgttaccg	22	
RNAi	PDKF	ttcccaactgtaatcaatcc	20	622
pRNAiACO1	PDKR	tgacaagtgatgtgtaagac	20	
pRNAiACO2	pRNAiACO1F	cttctttctacaaccccagc	20	350
pRNAiCACO	pRNAiACO1R	gacaccgttttcccacact	19	
	pRNAiACO2F	tctactgtaactcctggtgc	20	317
	pRNAiACO2R	caccaaaggccactagacag	20	
	pRNAiCACOF	atgaaggagtttgcagtggg	20	340
	pRNAiCACOR	ccgttagtaatcagctcaag	20	

**Table 4:** Effects of De Fossard and MS basal agar media on rooting of putative transgenic papaya shoots.

Treatment IBA (*μ*M)	Total number of shoots used	Rooting percentage (%)	Days to visible root	Number of primary roots	Mean roots length (cm)	Number of leaves abscised^3^
4.9 (DF)	180	22.0	40	0.8^c^	0.69^d^	1.1^c^
4.9 (MS)	60	46.0	30	2.2^a^	1.32^b^	1.1^a^
9.8 (MS)	60	50.0	21	2.5^a^	2.21^a^	1.0^a^
14.8 (MS)	60	12.0	60	0.9^b^	0.95^c^	1.3^a^

Means with the same letters are not significantly different at *P* < .05.

**Table 5:** Effects of vermiculite and perlite supplementations on roots development of putative transgenic papaya shoots.

Rooting medium	Total number of shoots used	Rooting percentage (%)	Days to visible root	Number of primary roots	Mean roots length (cm)	Number of leaves abscised
Vermiculite + 1/2 MS (sterile)	60	92.5	10	4.8^a^	4.16^a^	1.2^d^
Vermiculite + water (sterile)	60	50.0	23	2.1^c^	1.61^c^	4.7^a^
Perlite + 1/2 MS (sterile)	60	77.5	15	3.1^b^	2.56^b^	3.2^c^
Perlite + water (sterile)	60	47.5	22	1.2^d^	1.31^c^	5.1^a^
Vermiculite + water (nonsterile)	60	35.0	25	0.9^d^	1.09^d^	4.3^b^
Perlite + water (nonsterile)	60	23.8	26	0.8^d^	0.89^e^	5.2^a^

Means with the same letters are not significantly different at *P* < .05.

**Table 6:** Growth performance of transformed and nontransformed papaya plants 6 months after field planting.

Parameter	Putative transgenic plant (cm)	Control (nontransformed plant) (cm)
Plant height	181.78 ± 21.00	201 ± 25.00
Stem girth	18.12 ± 1.42	22 ± 1.23
Height of first flower	123.20 ± 9.21	185 ± 7.9
Height of first fruit	123.20 ± 9.21	185 ± 7.9
Mean internode length	2.10 ± 0.22	2.56 ± 0.90

Data is presented as a mean of 60 transgenic papaya plants and 10 control untransformed plants with ± standard deviation.
